# The p38 signaling pathway mediates quiescence of glioma stem cells by regulating epidermal growth factor receptor trafficking

**DOI:** 10.18632/oncotarget.16741

**Published:** 2017-03-31

**Authors:** Akio Soeda, Justin Lathia, Brian J. Williams, Qiulian Wu, Joseph Gallagher, Andreas Androutsellis-Theotokis, Amber J. Giles, Chunzhang Yang, Zhengping Zhuang, Mark R. Gilbert, Jeremy N. Rich, Deric M. Park

**Affiliations:** ^1^ Department of Neurosurgery, Gifu University, Gifu, Japan; ^2^ Department of Stem Cell Biology and Regenerative Medicine, Cleveland Clinic Foundation, Cleveland, OH, USA; ^3^ Department of Neurosurgery, University of Louisville, Louisville, KY, USA; ^4^ Division of Stem Cell Biology, Technische Universitat Dresden, Dresden, Germany; ^5^ Neuro-Oncology Branch, National Cancer Institute, NIH, Bethesda, MD, USA

**Keywords:** glioma, cancer stem cell, p38 MAPK, EGFR, quiescence

## Abstract

EGFR pathway is upregulated in malignant gliomas, and its downstream signaling is important for self-renewal of glioma cancer stem-like cells (GSC). p38 mitogen-activated protein kinase (MAPK) signaling, a stress-activated signaling cascade with suppressive and permissive effects on tumorigenesis, can promote internalization and ubiquitin ligase mediated degradation of EGFR. In this study, we investigated the role of p38 MAPK signaling on the self-renewal of GSCs with the hypothesis that inhibition may lead to enhanced self-renewal capacity by retention of EGFR. Inhibition of p38 MAPK pathway led to increase in EGFR expression but surprisingly, reduced proliferation. Additional functional evaluation revealed that p38 inhibition was associated with decrease in cell death and maintenance of undifferentiated state. Further probing the effect of p38 inhibition demonstrated attenuation of EGFR downstream signaling activity in spite of prolonged surface expression of the receptor. *In vitro* observations were confirmed in xenograft *in vivo* experiments. These data suggest that p38 MAPK control of EGFR signaling activity may alter GSC cell cycle state by regulating quiescence and passage into transit amplifying state.

## INTRODUCTION

Glioblastoma (GBM) is an incurable brain cancer with a median survival of approximately one year with multimodal treatment consisting of surgery, irradiation, and chemotherapy [1]. Accumulating observations suggest that GBM may be initiated and maintained by tumor cells with stem-like properties of self-renewal and multipotency [2–4]. These malignant glioma-derived cancer stem-like cells (GSC) are able to efficiently recapitulate the original tumor in the brains of immunodeficient mice, demonstrating the tumor initiating capacity and self-renewal [5]. Because GSC have been implicated in tumor initiation and therapeutic resistance, curative cancer treatments may require eradicating this subpopulation of the bulk tumor [6–9]. One potential strategy to specifically target the GSC population is to identify essential signaling pathways that regulate survival and proliferation. It has been reported that GSC demonstrate exquisite sensitivity to inhibition of the Akt pathway, a serine/threonine-specific protein kinase that regulates cellular growth-survival pathways downstream of receptor tyrosine kinases [10]. One such example of a receptor tyrosine kinase that is frequently altered in a variety of cancers including GBM is the epidermal growth factor receptor (EGFR), a member of the ErbB family of receptor tyrosine kinases [11]. Binding of the epidermal growth factor (EGF) to EGFR leads to receptor dimerization, autophosphorylation, and activation of downstream signaling cascades that regulate cell proliferation, motility, and survival [12]. A key tumorigenic event in the pathogenesis of GBM consists of activating disruption of the *EGFR* by mutation and amplification [13].

The p38 mitogen-activated protein kinase (MAPK) is a member of the serine/threonine kinase family that converts external stimuli to internal signaling events triggered by cellular stress, including exposure to ultra violet light, osmotic shock, inflammatory response, and heat shock [14, 15]. p38 signaling leads to suppression of cellular proliferation, and activation of apoptotic and senescence programs. Animal studies show that interference of the p38 pathway can have apparent contradictory effects such as proliferation and impaired differentiation of progenitor cells, and suppression of tumorigenicity [16, 17]. In contrast, p38 activation results in impaired self-renewal of hematopoietic stem cells [18]. Because the p38 pathway is often disrupted in human cancers, *p38* is increasingly being viewed as a tumor suppressor gene [19, 20]. One potential mechanism by which the p38 pathway may exert its tumor suppressive role is promoting internalization and degradation of the ligand bound EGFR [21–24].

We previously showed that EGFR signaling enhances the self-renewal capacity of GSC [25]. In this study we investigated the role of p38 MAPK pathway on the regulation of GSC self-renewal with the hypothesis that p38 MAPK pathway inhibition will lead to expansion of GSC through increased proliferation, maintenance of the undifferentiated state, and protection from apoptosis, resulting from enhanced EGFR signaling. Here we show that p38 pathway inhibition leads to overall increase in the number of GSC although the total number of mitotic events decreases; the result of a decrease in the rate of apoptosis. As hypothesized, we found that p38 pathway inhibition led to maintenance of the undifferentiated phenotype and decreased cell death, and p38 pathway activation was associated with spontaneous differentiation and increased apoptotic events. However, inhibition of p38 led to a decrease in both *in vitro* and *in vivo* GSC proliferation. Our data suggest that the p38 pathway affects survival, cell cycle state, and differentiation status of GSC by regulating EGFR trafficking.

## RESULTS

### GSCs demonstrate basal activation of the p38 MAPK pathway

**Figure 1:**
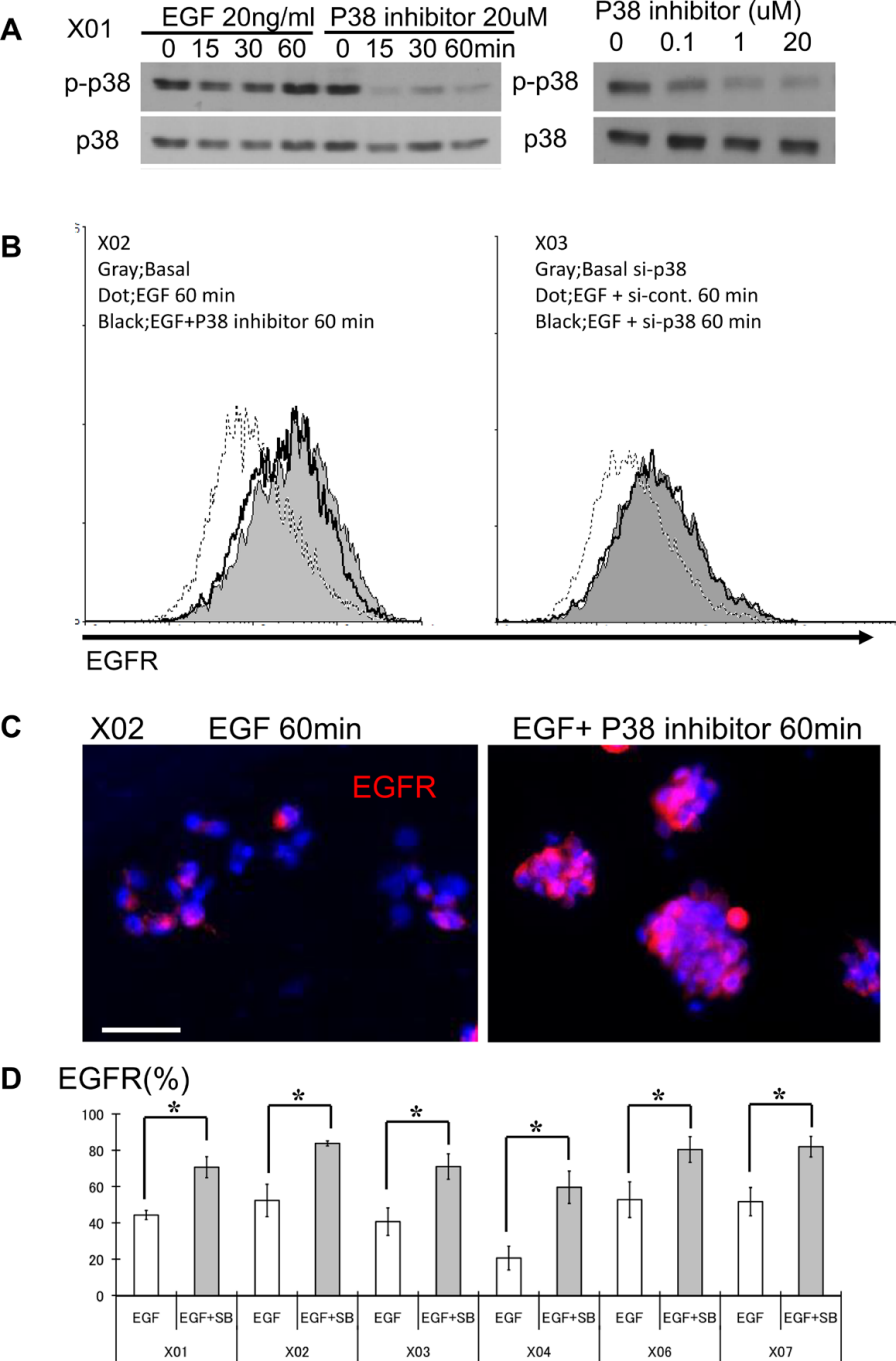
The p38 signaling pathway is activated in GSC and its inhibition leads to increase in surface expression of EGFR (**A**) GSC propagated with and without recombinant EGF were subjected to Western blot analysis for total and phospho-p38. GSCs were also treated with SB203580, an inhibitor of p38, at different time points and doses. (**B**) FACS analyses were performed with GSC at three different conditions: immediately prior to addition of EGF (20 ng/ml), 60 minutes after exposure to EGF, and 60 minutes after treatment with EGF and SB203580. FACS histograms show rapid reduction (approximately 60%) in the expression level of surface EGFR from baseline (solid gray) to addition of EGF (dotted line). This reduction is abrogated in the presence of the p38 inhibitor (10 μM SB203580) (black line). (**C**) Immunocytochemical staining was performed with an antibody directed against the extracellular domain of the EGFR. GSC exposed to the p38 inhibitor (10 μM SB203580) show higher surface expression. Bar = 50 μm. (**D**) Six distinct GSC lines were propagated in the presence of EGF (20 ng/ml), with and without the p38 inhibitor (10 μM) and the surface expression level of EGFR was determined by FACS analysis. All GSC lines revealed increase in EGFR expression in the presence of p38 inhibitor. The results shown in the graph are mean + S.D. from three experiments. *p < 0.05.

All experiments were performed with nine malignant-glioma derived GSC lines (7 glioblastomas: X01, X02, X04, X05, X06, 08–322, 08–387, 1 gliosarcoma: X07, and 1 anaplastic oligoastrocytoma: X03) established from acutely resected surgical specimens under a protocol approved by the Institutional Review Board. The GSC lines demonstrate extensive self-renewal as assessed by sphere-forming assay, a surrogate marker, are multipotent with the capacity to differentiate into neuronal and glial lineages, and express nestin, sox2, and CD133; all markers of the undifferentiated phenotype. Transplantation of these GSC lines into the brains of immunodeficient mice recapitulated the original tumor (Supplementary Figure 1) [25, 26]. By immunoblotting, we found basal activation of the p38 MAPK pathway in GSC; the level of p38 activation did not change with addition of exogenous EGF suggesting that the basal activation state of p38 is not regulated by mitogenic signaling (Figure 1A and Supplementary Figure 3). To determine the feasibility of modulating the p38 signaling pathway in GSC, we used pharmacologic agents to repress (SB203580, inhibitor of p38 α/β isoforms) and activate (anisomycin) the p38 signaling pathway. SB203580 inhibited the p38 signaling pathway in a dose concentration-dependent manner (Figure 1A). Similar results were observed in the other GSC lines used in these experiments.

### Ligand bound EGFR complex expression is sustained with p38 inhibition

Ligand bound EGFR complex is internalized for recycling or degradation [27]. We used an EGFR antibody that recognizes the extracellular domain of EGFR to determine the effect of p38 inhibition on EGFR fate with addition of recombinant EGF. The FACS histograms show approximately 60% reduction in the fluorescence intensity of EGFR receptor expression within 60 minutes of exposure to EGF as determined by measuring the change in fluorescence intensity (Figure 1B). Pharmacologic inhibition of p38 results in sustained EGFR surface expression (Figure 1B, left). Because drugs can have unintended pleiotropic effects, we used a genetic approach to transiently knockdown p38 expression and observed a similar effect (Figure 1B, right). Immunocytochemistry also revealed greater EGFR surface expression with p38 inhibition (Figure 1C). Although the basal expression level of EGFR was different among the examined GSC, we found a consistent pattern of sustained EGFR surface expression upon treatment with recombinant EGF with p38 inhibition (Figure 1D).

**Figure 2:**
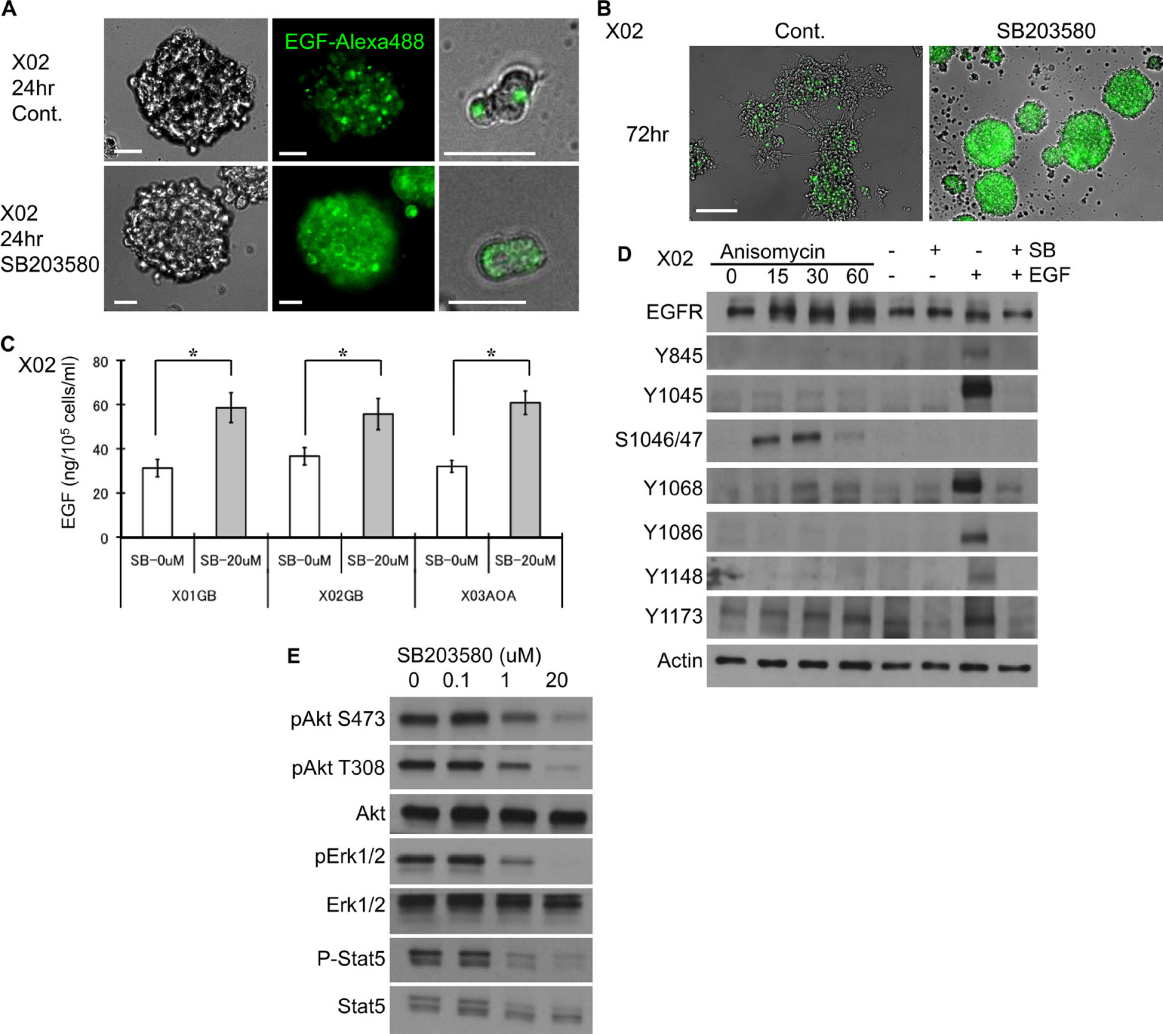
EGF-EGFR complex expression is sustained with p38 inhibition (**A**) Recombinant EGF protein labeled with a fluorophore was used to follow the fate of the EGF-EGFR complex in untreated and treated (10 μM SB 203580) GSC and imaged 24 hours after treatment. Images show higher overall expression of the dye (Alexa 488) that is largely localized to the cell surface suggesting decreased internalization and degradation of the complex upon inhibition of the p38 pathway. Bar = 20 μm. (**B**) The same experiment performed at 72 hours demonstrates a greater difference in the preservation of the dye-labeled EGF. Bar = 100 μm. (**C**) Untreated control and treated (10 μM SB203580) GSC were exposed to EGF (100 ng/ml) at day 1, and ELISA for EGF in the supernatant was performed at day 3. Inhibition of p38 results in higher EGF in the supernatant, implying decreased consumption. The results shown in the graph are mean + S.D. from three experiments. *p < 0.05. (**D**) EGFR phosphorylation status was evaluated by Western blotting on GSC subjected to alteration of the p38 pathway. GSC were treated with anisomycin or SB203580 with and without EGF (20 ng/ml, 15 minutes). The integrity of the EGFR apparatus is indicated by phosphorylation of key tyrosine residues upon addition of recombinant EGF, an effect that was antagonized by SB203580 (right two lanes). Addition of anisomycin (30 μM) led to phosphorylation of serine 1046/47, an event that is known to be associated with subsequent degradation of EGFR. (**E**) The signaling events downstream of EGFR were evaluated by Western blotting of GSC treated with and without SB203580. Inhibition of p38 pathway was associated with reduced activation of Akt, ERK, and STAT5 in a dose-dependent manner.

To confirm that the EGFR surface expression that was prolonged by p38 inhibition is ligand bound, we followed the fate of EGF-EGFR complex using a fluorophore-labeled recombinant EGF. Immunocytochemical staining of a representative GSC shows that control cells internalize and degrade the complex within 24 hours (Figure 2A). However, inhibition of the p38 pathway leads to surface retention of the EGF-EGFR complex (Figure 2A). Preservation of the ligand bound EGFR complex is seen even at 72 hours (Figure 2B). Furthermore, p38 inhibition results in decreased EGF consumption as determined by ELISA performed on the supernatant of the untreated and treated GSC (Figure 2C). These data suggest that inhibition of p38 pathway delays EGF-EGFR complex internalization and degradation, decreases EGF consumption, and attenuates EGFR turnover.

### Modulation of the p38 pathway impacts EGFR activation and its downstream signaling cascade

Upon binding of the ligand to EGFR, the tyrosine 1045 site on the cytoplasmic domain of the EGFR becomes autophosphorylated. This leads to phosphorylation of the Cbl ubiquitin ligase and subsequent ubiquitination [24, 28]. To determine the integrity of EGFR signaling pathway and effect of manipulating the p38 pathway, we performed a series of immunoblotting experiments. Addition of recombinant EGF led to phosphorylation of tyrosine 845, 1045, 1068, 1086, 1148, and 1173 (Figure 2D, lane SB− and EGF+). All are autophosphorylation sites; save for tyrosine 845, which is activated by Src [12]. Inhibition of the p38 pathway even in the presence of EGF showed attenuation of activation at all sites (Figure 2D, lane SB+ and EGF+). Pharmacologic activation of the p38 pathway led to phosphorylation of serine 1046/47 residues (Figure 2D, lanes Anisomycin), sites that are critical for degradation of EGFR [29]. We next investigated the effect of p38 inhibition on EGFR downstream signaling cascade. Although p38 inhibition led to prolonged surface expression of EGF-EGFR complex, the EGFR downstream signaling activity was decreased (Figure 2E). We observed reduction in the activation level of Akt at both serine 473 and threonine 308 sites, ERK, and STAT5. These experiments suggest that in GSC, boosting p38 signaling may prime for EGFR degradation by phosphorylation of serine 1046/47, and inhibition of p38 leads to rescue of EGFR from ubiquitination and degradation by preventing phosphorylation of tyrosine 1045 and serine 1046/47. The reduced EGFR downstream events upon inhibition of p38 signaling may be due to attenuation of additional phosphorylated tyrosine residues.

### p38 pathway inhibition of GSC results in decreased proliferative activity and confers protection from apoptosis

**Figure 3:**
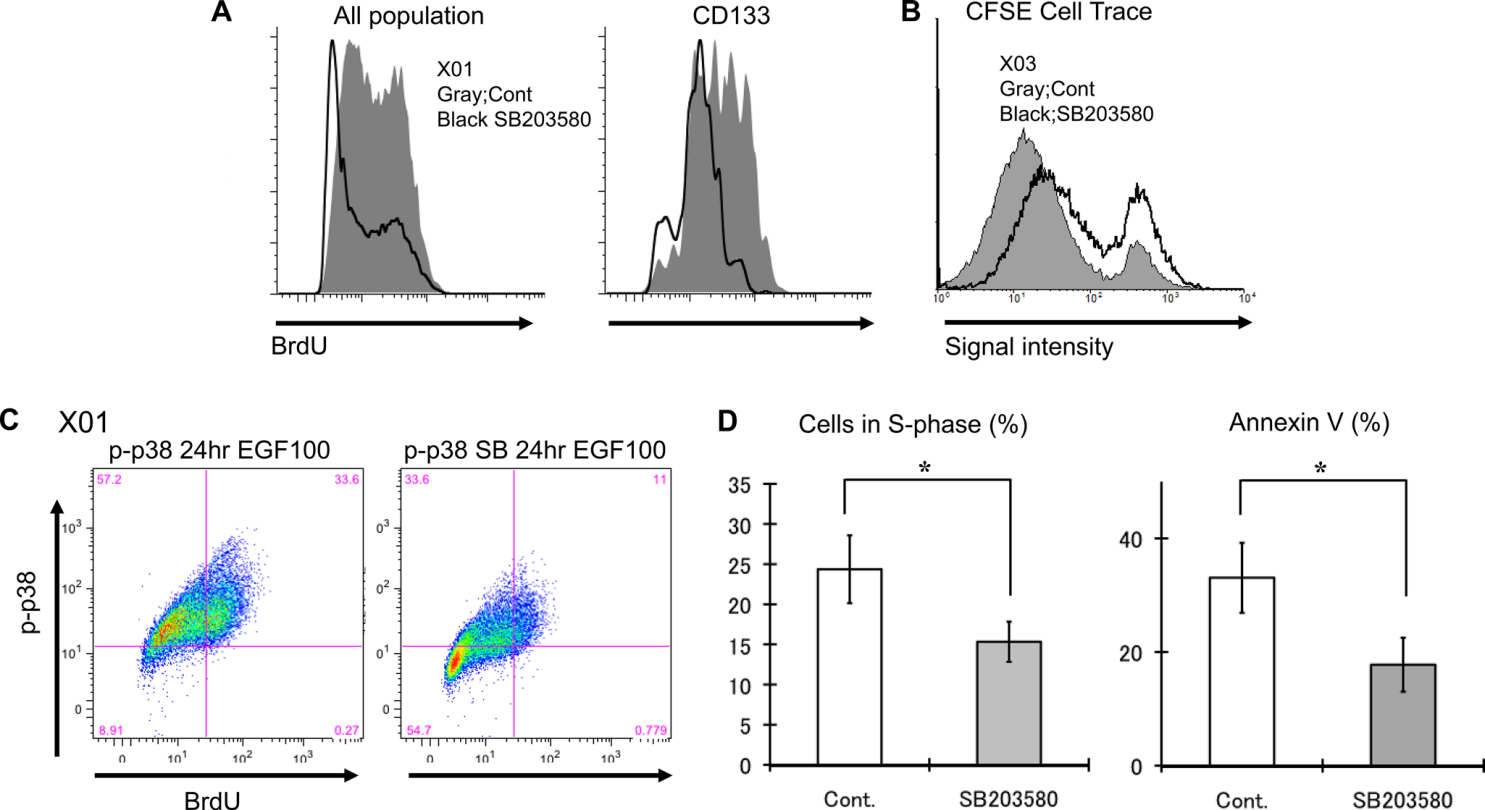
GSC inhibition of the p38 pathway suppresses proliferation and may confer protection from apoptosis (**A**) Untreated control and treated (10 μM SB203580) GSC were incubated for 3 hours with BrdU (10 μM) and expression level was assessed by FACS analysis. Similar experiments were performed with CD133-positive cells purified by cell sorting. Inhibition of the p38 pathway led to decreased BrdU incorporation in both bulk GSC population and CD133-positive subpopulation. (**B**) CFSE dye experiment was used to further corroborate the BrdU experiments. p38 inhibition (solid black line) resulted in greater fraction of cells retaining higher signal intensity, suggesting slower cell division rate. (**C**) This is a double labeling experiment for p-p38 and BrdU of untreated control GSC and treated (10 μM SB203580) analyzed by FACS demonstrating decreased incorporation of BrdU in population of cells with low p-p38 upon treatment with p38 inhibitor. (**D**) The cell cycle status of untreated control and treated (10 μM SB203580) GSC was determined by FACS analysis. p38 inhibition reduced the proportion of cells in the S-phase. Additionally the rate of apoptosis was evaluated by Annexin V immunopositivity and analyzed by FACS. p38 inhibition was associated with decreased apoptosis. The results shown in the graph are mean + S.D. from three experiments. *p < 0.05.

Inhibition of p38 has been reported to increase proliferation and attenuate apoptosis of non-neoplastic progenitor cells [16]. To determine the effect of p38 inhibition on proliferation and apoptosis of GSC, we performed a series of FACS experiments. GSCs treated with p38 inhibitor displayed decreased BrdU incorporation at 3 hour time frame (Figure 3A and Supplementary Figure 4). The same result was observed when the experiment was repeated using highly purified fraction of CD133-positive GSC. To confirm that the delay in BrdU incorporation was a reflection of decreased cell division, we used CFSE dye to track cell divisions. Cell division leads to dilution of the fluorescence signal intensity that can be measured by FACS. Analysis of CFSE fluorescence at 48 hours shows p38 inhibition results in greater proportion of GSC that have not divided (Figure 3B). FACS analysis was further used to determine the basal state of p38 activation in relation to cell cycle. BrdU incorporation was predominantly seen in p-p38 positive GSCs (Figure 3C, left). However, exposure to p38 inhibitor led to reduction of BrdU/p-p38 positive population and increase of BrdU/p-p83 negative cells (Figure 3C, right) suggesting that the activation state of p38 directly correlates with cell cycling. This observation is further supported by data showing p38 inhibition leads to a reduction of GSC in the S-phase of the cell cycle (Figure 3D). To investigate the effect of p38 inhibition on cell death, we assessed Annexin V immunoreactivity via FACS. We observed reduction in the number of cells labeling for Annexin V upon p38 inhibition (Figure 3D), that was not due to difference in cell size (data not shown). These experiments suggest that GSC p38 inhibition leads to decrease in proliferative activity (Supplementary Figure 2) and protection from apoptosis.

### p38 pathway inhibition of GSC maintains stemness by preventing differentiation

**Figure 4:**
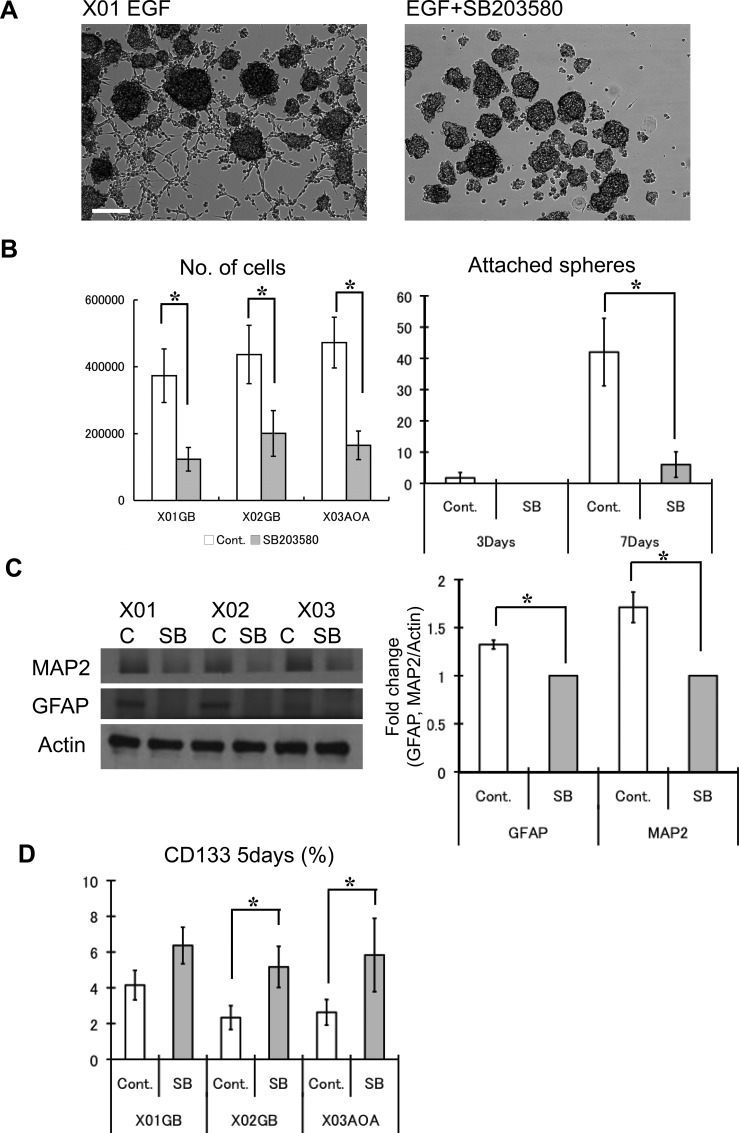
Inhibition of the p38 pathway maintains stemness of GSC and attenuates differentiation (**A**) Untreated control and treated (10 μM SB203580) GSC were supplemented with EGF (100 ng/ml at day 1) and incubated for 7 days. This picture is a representative photomicrograph of the X01 culture quantified in Figure 4B. Bar = 100 μm. (**B**) The respective numbers of cells was determined by Tryphan exclusion. At 3 days, untreated control cells began to attach to the substratum, which increased dramatically by day 7. The results shown in the graph are mean ± S.D. from three experiments. (**C**) Western blots were performed on untreated control and treated (10 μM SB203580) GSC for glial (GFAP) and neuronal (MAP2) lineage markers. Levels of GFAP and MAP2 were normalized to actin and expressed as mean fold change. The results shown in the graph are mean ± S.D. from three experiments. *p < 0.05. (**D**) The relative abundance of the CD133-positve cells was determined by FACS analysis for each condition. The proportion of CD133 positive cells increased with addition of p38 inhibitor. The results shown in the graph are mean ± S.D. from three experiments. *p < 0.05.

To determine the effect of p38 inhibition on the self-renewal activity of GSC, we used sphere-forming assay, an *in vitro* determinant of self-renewal capacity of CSC [25, 30]. Within 3 days of incubation (EGF was given only on day 1), cell number was greater for untreated GSCs compared to GSCs treated with the p38 inhibitor (Figure 4A–4B). This was anticipated considering the previous experiments on cell cycle state that demonstrated a reduction in mitosis. In addition, we observed a greater number of control cells attaching to the surface, a sign of spontaneous differentiation. Without p38 inhibition, the number of attached spheres increased strikingly by day 7, and the expression of GFAP and MAP2, markers of glial and neuronal lineages, was greater (Figure 4B–4C). p38 inhibition of GSC led to enhanced retention of cells with surface expression for CD133 (Figure 4D). These observations suggest that while p38 inhibition of GSC leads to diminished proliferative activity, the tumor cells maintain undifferentiated GSC state by blocking differentiation into terminal lineages.

### p38 pathway activation leads to decreased EGFR expression and reduction of GSC tumorspheres

**Figure 5:**
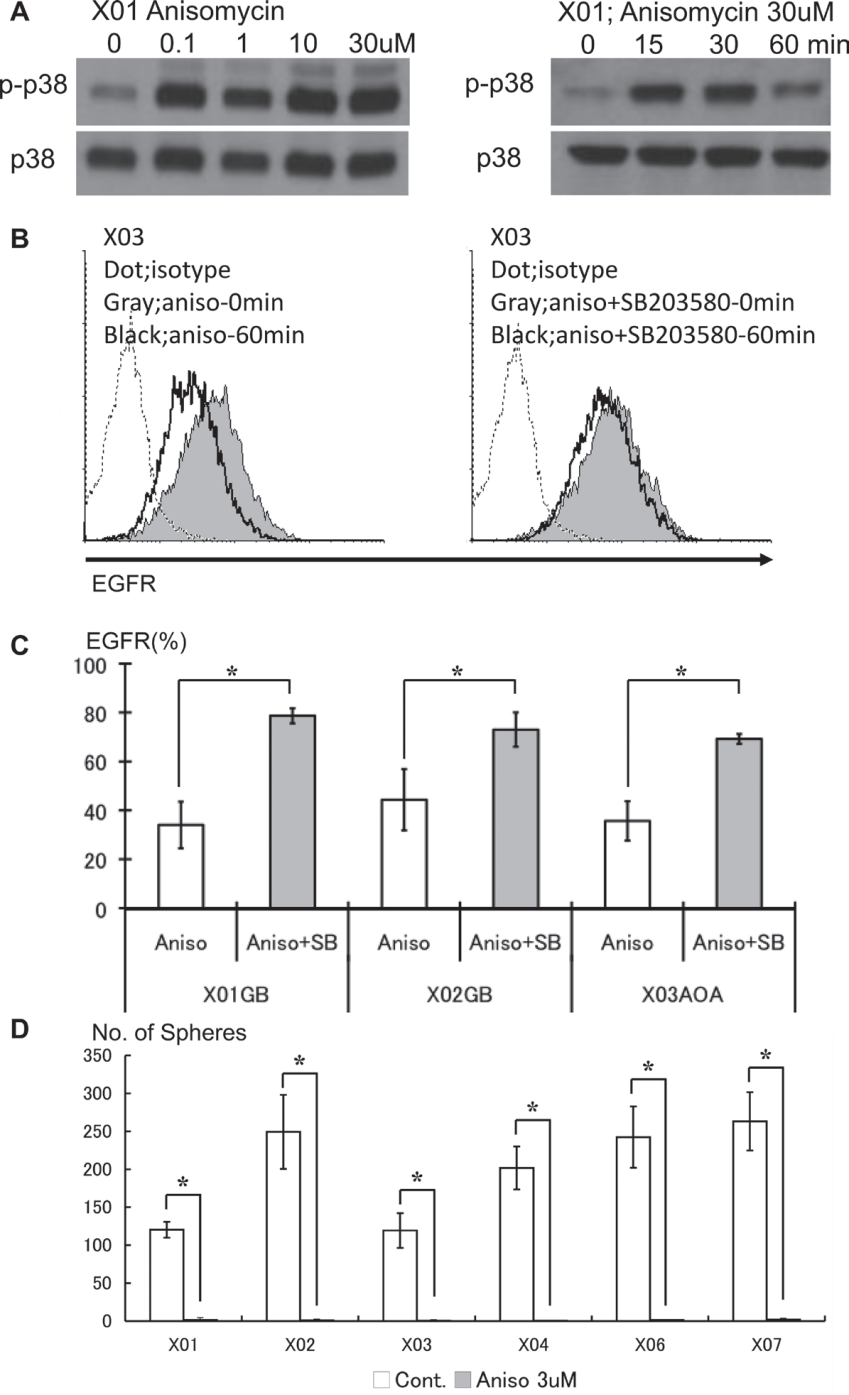
Pharmacologic activation of the p38 pathway leads to decreased EGFR and poor self-renewal capacity of the GSC (**A**) GSC treated with anisomycin, a p38 activator, were evaluated by Western blotting. Anisomycin enhances the phosphorylation state of p38 in a dose-dependent manner. (**B**) Untreated control (solid gray) and GSC exposed to anisomycin (10 μM) for 1 hour were analyzed for expression of EGFR by FACS (left). The reduced expression of EGFR seen with anisomycin treatment was abrogated with addition of SB203580 (10 mM). (**C**) The reversal of EGFR expression upon p38 inhibition by SB203580 on GSC treated with anisomycin was observed in all lines studied. Data from three representative lines are shown. The results shown in the graph are mean ± S.D. from three experiments. *p < 0.05. (**D**) The *in vitro* self-renewal capacity of untreated control and GSC exposed to anisomycin (3 μM for 48 hours) was assessed by sphere-forming assay. Activation of the p38 pathway led to striking reduction of self-renewal. The results shown in the graph are mean + S.D. from three experiments. *p < 0.05.

GSC show basal activation of the p38 signaling pathway. To investigate the effect of further amplifying activation state of the p38 pathway on proliferation, differentiation, and cell death, we used anisomycin, a selective p38 MAPK activator [23, 24]. We validated the p38 activating effect of anisomycin on the GSC lines. Anisomycin was capable of further boosting the basal p38 activation level in a dose concentration-dependent manner (Figure 5A). Pharmacologic activation of p38 pathway on the GSC led to in reduction of EGFR surface expression, an effect partly abrogated by addition of the p38 inhibitor (Figure 5B–5C). We observed that excess activation of the p38 pathway resulted in striking attenuation of sphere-forming capacity of the GSC (Figure 5D). These data show that unopposed activation of the p38 pathway leads to reduction of surface EGFR and loss of *in vitro* self-renewal capacity of GSC.

### p38 inhibition delays tumorigenicity of GSC while maintaining robust EGFR expression and tumor cell viability

**Figure 6:**
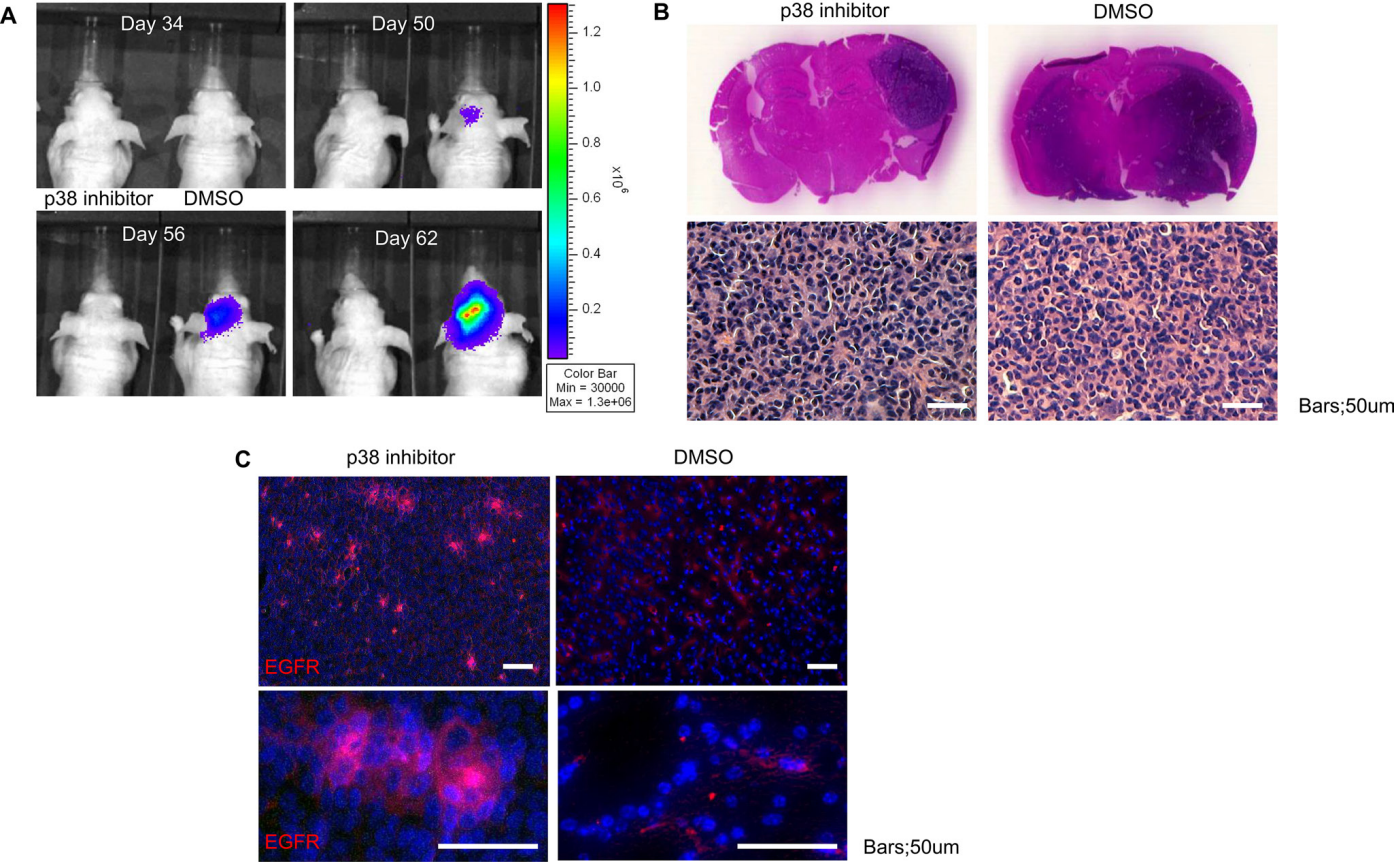
Inhibition of p38 delays *in vivo* tumor growth but increases EGFR expression (**A**) Tumor size was monitored via bioluminescence for mice treated with DMSO or p38 inhibitor demonstrating slow tumor growth (ten mice per group). (**B**) Post-mortem examination of coronal sections stained with hematoxylin and eosin (H&E) of mice treated with DMSO or p38 inhibitor shows larger tumor volume in the control group as well as more diffuse infiltration. Representative microscopic sections from control and p38 inhibitor treated groups demonstrates the typical histological appearance of glioblastoma with significant atypia and nuclear pleomorphism. (**C**) Immunofluorescent staining for EGFR (Alexa 555) counterstained with DAPI demonstrates increased EGFR expression in the p38 inhibitor group compared to control. Bars = 50 um.

To assess the role of p38 inhibition on modulation of tumor formation, we investigated the *in vivo* effect of p38 inhibition on GSC. Inhibition of the p38 signaling pathway in transplanted GSC led to delay in tumor development (Figure 6A). Histological studies revealed p38 treatment led to viable, but smaller overall tumor (dark blue stained cells on coronal sections), that did not infiltrate into surrounding tissues (Figure 6B). Additional analysis of the tissue sections for EGFR revealed enhanced expression of EGFR compared to control animals (Figure 6C). The *in vivo* data confirms the *in vitro* observations and suggest that inhibition of p38 results in viable tumor cells but with relative cell cycle arrest.

## DISCUSSION

Better understanding of the signaling pathways that regulate proliferation, differentiation, and cell death of cancer cells is necessary to identify more effective therapeutic approaches. Given previous reports demonstrating interaction of EGFR and p38 MAPK pathways, and the importance of EGFR signaling on the self-renewal of GSC, we investigated the role of p38 pathway in GSC. We found basal activation of p38 pathway in all of the examined GSC lines. Inhibition of the p38 pathway led to decrease in overall proliferation, but increase in undifferentiated GSC population potentially from reduction in differentiation and apoptotic events. p38 inhibition of tumor xenografts showed viable cells with robust EGFR expression, but at a state of relative growth and migration arrest. Unopposed activation of the p38 pathway in the GSC resulted in striking attenuation of self-renewal capacity.

### p38 pathway regulates GSC self-renewal and differentiation

p38, a member of the MAPK family along with the extracellular signal-regulated kinase (ERK) and c-Jun N-terminal kinase (JNK) pathways, plays an important role in responding to cellular stress and inflammation. Because activation of the p38 pathway results in negative regulation of cellular proliferation and induction of cell death programs, it is increasingly being recognized as a potential tumor suppressor [20]. Inhibition of the p38 pathway is associated with enhanced cellular growth. Conversely, activation of p38 is associated with loss of stem cell self-renewal [18, 31]. We therefore investigated how the p38 pathway may regulate the self-renewal capacity of GSC, a population of malignant stem cells. Pharmacologic activation of the p38 pathway with anisomycin, an antibiotic isolated from *Streptomyces griseolus* that inhibits protein synthesis by blocking peptidyl transferase activity in eukaryote ribosomes, was associated with rapid attenuation of EGFR surface expression. Further probing of this process revealed phosphorylation of EGFR serine 1046/47 residues, an event believed to trigger degradation of the receptor [29]. GSC treated with anisomycin exhibited almost complete loss of self-renewal activity. Increased p38 MAPK signaling activity is linked to activation of apoptosis. Because dead cells are unable to self-renew, the loss of self-renewal capacity of GSC upon p38 activation may reflect simply an effect on cell death. As we have shown that EGFR signaling is critical to maintenance of GSC self-renewal capacity, another potential explanation may be that by promoting internalization of EGFR, the GSC may lose the signaling apparatus required for self-renewal [25]. EGFR is an attractive target for many types of cancer. In addition to attenuating its signaling activity by antibodies directed against the ligand binding domain or small molecule inhibitors of the tyrosine kinase activity, programming the receptor for degradation by modulating the p38 pathway may offer an alternative therapeutic strategy.

The critical involvement of the p38 pathway during embryonic development suggests that it may play an important role in induction of differentiation programs [32, 33]. As suspected, disruption of p38 results in impaired differentiation of non-neoplastic stem-progenitor cells [16, 17]. We investigated the role of p38 signaling on the maintenance GSC differentiation state. GSC were supplemented with exogenous EGF, a growth factor necessary to prevent differentiation, only on day 1 and then evaluated at day 7. Control tumorspheres differentiated into glial and neuronal lineages as expected with EGF withdrawal, while GSC subjected to p38 inhibition retained undifferentiated phenotype even without EGF. With p38 inhibition, we observed an overall increase in the number of undifferentiated GSC. Because in animal models, GSC appear to possess enhanced tumorigenicity and resistance to irradiation, it is believed that eradication of GSC population is necessary for delivery of effective and durable treatments [5, 34]. To target the cancer stem cell compartment of the bulk glioma, a variety of strategies have been proposed, including differentiation-inducing approaches and inhibition of developmental-stem cell signaling pathways such as notch and hedgehog. Here we observed that activation of the p38 MAPK signaling network led to decreased EGFR expression and reduction of GSC tumorspheres suggesting therapeutic opportunities in concert with chemoradiation. The p38 MAPK signaling pathway may be a possible mechanism to regulate differentiation of GSC.

### p38 inhibition reduces the proliferative capacity of GSC

The tumor suppressive effects of p38 MAPK has been attributed to its negative regulation on cell proliferation. Therefore, in most cases inhibition of the p38 pathway leads to adoption of pro-proliferation bias [17, 20]. In contrast, we consistently found that inhibition of the p38 pathway led to diminished proliferative activity of the GSC, in spite of increase in surface expression of EGFR. Although it is well established that p38 regulation of cellular activity is cell type-specific [35], we observed that inhibition of p38 led to increased EGFR expression and maintenance of the undifferentiated state, but consistently reduced proliferation in all of the examined GSC. Close examination of the EGFR phosphorylation state offers a potential explanation. Addition of EGF led to phosphorylation tyrosine residues 845, 1068, 1086, 1148, 1173, and 1045. Tyrosine 845, located in the kinase domain, is phosphorylated by Src and functions to stabilize the activation loop that is critical for transmission of mitogenic signals [36]. Phosphorylation of tyrosine residues 1068, 1086, 1148, and 1173 allows for interaction with proteins containing the Src homology 2 domains (SH2) such as GRB, PLCγ, and Shc [36–38]. Recruitment of these additional signaling proteins leads to activation of downstream pro-growth pathways. Phosphorylation of tyrosine 1045 primes the receptor for binding of ubiquitin ligase Cbl and eventual destruction [39, 40]. p38 inhibition interrupted phosphorylation of all these sites. Interference at tyrosine 1045 is consistent with prolonged surface expression of EGFR. Despite the persistent expression of the receptor, disrupted phosphorylation at the remaining tyrosine sites (845, 1068, 1086, 1148, 1173) attenuates the kinase activity and impairs recruitment of additional signaling proteins to the receptor. This may result in a net effect of expression of a receptor, but without the ability to signal and activate downstream pathways. This proposed mechanism is in agreement with the observation that the activation states of Akt, Erk, and STAT5 were attenuated with p38 inhibition in a dose-dependent manner.

The combination of increased EGFR expression, decreased downstream signaling of EGFR, reduced proliferation, and maintenance of the undifferentiated state, all resulting from inhibition of the p38 pathway is suggestive of a quiescent state. Although quiescence is a defining feature of regenerative stem cells until specifically activated to divide [41], cancer stem cells have largely demonstrated uncontrolled proliferation. Such observations have led to suggestions that expression of stem cell markers on brain cancer cells may merely label cells with higher proliferative potential [7, 42]. Yet, the presence of basal activation of the p38 pathway in GSC that can be modulated to influence proliferation and differentiation may indicate presence of a quiescence program. The interpretation can also be expanded to indicate that p38 pathway inhibition promotes maintenance of GSC stemness by blocking the passage into a transit amplifying state. During development and wound repair, non-neoplastic stem cells generate transit amplifying cells that are characterized by an active proliferative state prior to giving rise to differentiated and highly specialized effector cells [43, 44]. Our observations in this paper suggest that similar mechanisms may operate in cancer stem cells from glioblastoma multiforme, and distinguish between effects on self-renewal and proliferative properties. Because non-dividing cancer cells are more likely to be resistant to therapies, perturbation of the p38 network may provide new therapeutic opportunities.

## MATERIALS AND METHODS

### Cell culture and pharmacologic agents

Tumorsphere cultures were performed as described previously, using DMEM-F12 (GIBCO-Invitrogen, La Jolla, CA), penicillin G, streptomycin sulfate, B-27 without vitamin A (GIBCO-Invitrogen), and recombinant human EGF (20 ng/ml; R&D Systems, Minneapolis, MN) [26, 45]. Cells were cultured in HERAcell incubators (Thermo Electronic Corporation, Asheville, NC) at 37°C, ≥ 95% relative humidity, and 5% CO_2_ with 20% O_2_environment. SB203580, a highly selective inhibitor of the p38α (MAPK14) and β (MAPK11) isoforms, and anisomycin were purchased from EMD Biosciences (San Diego, CA).

### Flow cytometry (FACS) analysis

Populations of CD133 (putative marker of GSC) and EGFR positive cells were evaluated on a Coulter EPICS cytometer (Beckman Coulter, Fullerton, CA) as described previously [46]. Each sample was labeled with phycoerythrin (PE)-conjugated anti-human EGFR (EGFR.1) antibody (BD Biosciences, San Jose, CA) or PE-conjugated anti-CD133/1 (AC133) antibody (Miltenyi Biotec, Auburn, CA) according to the manufacturers’ recommendations. For BrdU labeling of proliferating cells *in vitro*, BrdU was added to tumorspheres at a final concentration of 10 μM 3 hrs prior to fixation. BrdU uptake was detected with a FITC BrdU Flow kit (B44, BD Biosciences) on dissociated single cells and analyzed with/without PE-conjugated anti-CD133/1 antibody (Miltenyi Biotec) anti-phospho-p38 MAPK (3D7, Cell Signaling Technology) according to the manufacturer's recommendation. CellTrace carboxyfluorescein diacetate succinimidyl ester (CFSE) cell proliferation kit (Molecular Probes, Eugene, OR) and Annexin V-FITC apoptosis detection kit (BD Biosciences) were used according to manufacturers’ protocol. Appropriate compensation and isotype controls were used. All experiments were performed in triplicate.

### Western blotting

Western blot analyses were performed as described previously [25]. The following antibodies were purchased from Cell Signaling Technology: p38 MAPK (#9212), phospho-p38 MAPK (Thr180/Tyr182, #9212), Microtubule-associated protein 2 (MAP2; #4542), glial fibrillary acidic protein (GFAP; #3670), EGF receptor (#2232), phospho-EGFR (Ser1046/1047, #2238; Tyr1045, #2237; Tyr1068, #2234; Tyr1086, #2220; Tyr1148, #4404; Tyr1173, #4407), Akt (#4685), phospho-Akt (Ser473, #9271; Thr308, #2965), Stat5 (#9363), phospho-Stat5 (Tyr694, #9359). Beta actin (C4) was from Santa Cruz Biotechnology. ERK1/2 (Cell Signaling Technology), phospho-ERK1/2 (Thr202/Tyr204, Cell Signaling Technology). Cells were lysed in buffer (20 mM Tris-HCl, pH 7.4, 150 mM NaCl, 1 mM EGTA, 1% Triton X-100, 2.5 mM sodium pyrophosphate, 1 mM β-glycerol phosphate, 1 mM Na_3_VO_4_, 1 μg/ml leupeptin, and 1 mM phenylmethylsulfonyl fluoride). After brief sonication, lysates were clarified by centrifugation at 12,000 × g for 10 min at 4°C, and protein content in the supernatant was measured according to the Bradford method. Aliquots (30–50 μg of protein per lane) of total protein were separated by 7.5–15% SDS-polyacrylamide gel electrophoresis and blotted onto nitrocellulose transfer membranes (0.2 μm; Amersham Biosciences, Buckinghamshire, UK). Each membrane was blocked with 5% non-fat dry milk in TBS-T (20 mM Tris-HCl, pH 7.6, 137 mM NaCl, and 0.01% Tween-20) for 1 hour at room temperature, followed by incubation with the appropriate primary antibodies overnight at 4°C. After extensive washing with TBS-T, each membrane was further incubated with horseradish peroxidase-conjugated anti-rabbit, anti-mouse, or anti-goat secondary antibodies (1:1,000) for 1 hour at room temperature in TBS-T containing 5% non-fat dry milk. Detection was performed using enhanced chemiluminescence reagent (Amersham Biosciences), and was quantified using ImageJ software (National Institutes of Health).

### Enzyme-linked immunosorbent (ELISA) assays

EGF protein levels were determined by ELISA performed with Quantikine immunoassay kit for human EGF (R&D systems) according to manufacturer's instructions as described previously [46]. 1 × 10^6^ GSC were transferred to T25 Falcon culture flasks with suspension medium containing 100 ng/ml EGF with/without SB203580. After 72 hr incubations, supernatants were used immediately after collection or frozen at −20°C until they were processed. All experiments were performed in triplicate.

### Indirect immunofluorescence microscopy

Immunocytochemistry was performed as described previously [9, 26]. Anti-EGFR antibody (1:500; Santa Cruz Biotechnology) used for detection of surface EGFR was probed with Alexa Fluor 555 secondary antibody (1:1,000; Molecular Probes). Cells were counterstained with 4′,6-diamidino-2-phenylindole (DAPI). To track the fate of ligand bound EGFR, Alexa Fluor 488 dye-labeled EGF complex (Molecular Probes) was used. The following hardwares were used: Zeiss Axiovert 200 microscope (Carl Zeiss, Gottingen, Germany), Plan-Neofluar 20× and 40× objectives, AxioCam MrM CCD camera. Axiovision software was used for image acquisition (Carl Zeiss).

### *In vivo* bioluminescence

All *in vivo* experiments were performed as described in accordance with Institutional Animal Care and Use Committee approval. GSCs with stably expressing firefly luciferase were intracranially injected (75,000 cells) into the right forebrains of 4–6 week old athymic nude mice. Mice were injected with luciferin (100 uL total volume of 30 mg/mL luciferin solution for 100 mg/kg dose) prior to imaging and evaluated using the Xenogen imaging system every four days prior to confirmation of tumor engraftment, after which imaging was done at least three times per week. Survival studies were performed as in our prior report with intracranial injection of lentivirally infected GSCs [47]. Mice were monitored daily until the development of neurological or constitutional signs (e.g., ataxia, lethargy, seizures) at which point they were sacrificed and brains were removed for histological analysis.

### Statistical analysis

Statistical differences were evaluated with Student's *t*-test. A *p*-value of less than 0.05 was considered statistically significant.

## SUPPLEMENTARY MATERIALS FIGURES AND TABLES


