# Supplementary material for: The p38 signaling pathway mediates quiescence of glioma stem cells by regulating epidermal growth factor receptor trafficking
Source: Oncotarget. 2017 Mar 31;8(20):33316–28. doi: 10.18632/oncotarget.16741 (PMC5464870; doi:10.18632/oncotarget.16741)
Supplement: Supplementary file 1 [file oncotarget-08-33316-s001.pdf]

The p38 signaling pathway effects quiescence of glioma stem cells by regulating epidermal growth factor receptor trafficking

Supplementary Materials

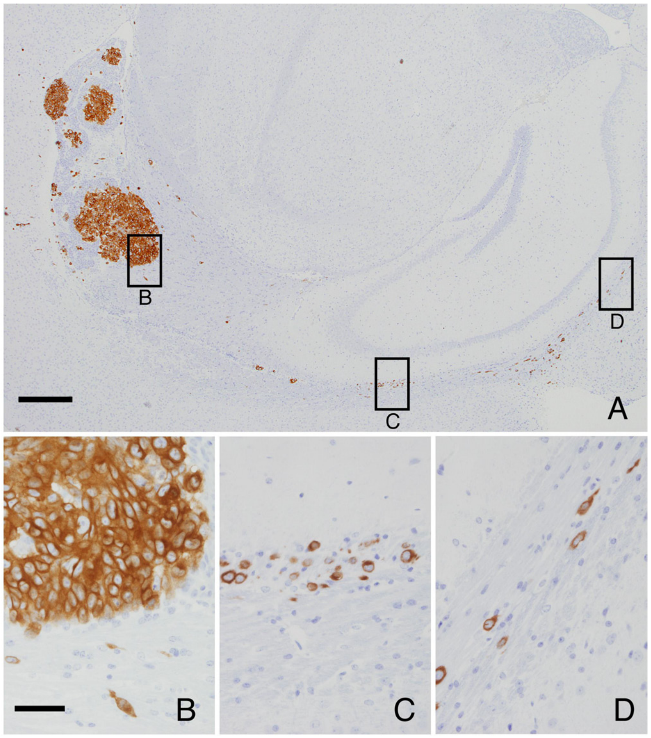

Scale bars, 500 μm in A; 50 μm in B

Supplementary Figure 1: Transplantation of GSC into immunodeficient mouse brain leads to formation of tumors with extensive infiltrative capacity. Mouse brain sections were stained for human EGFR.

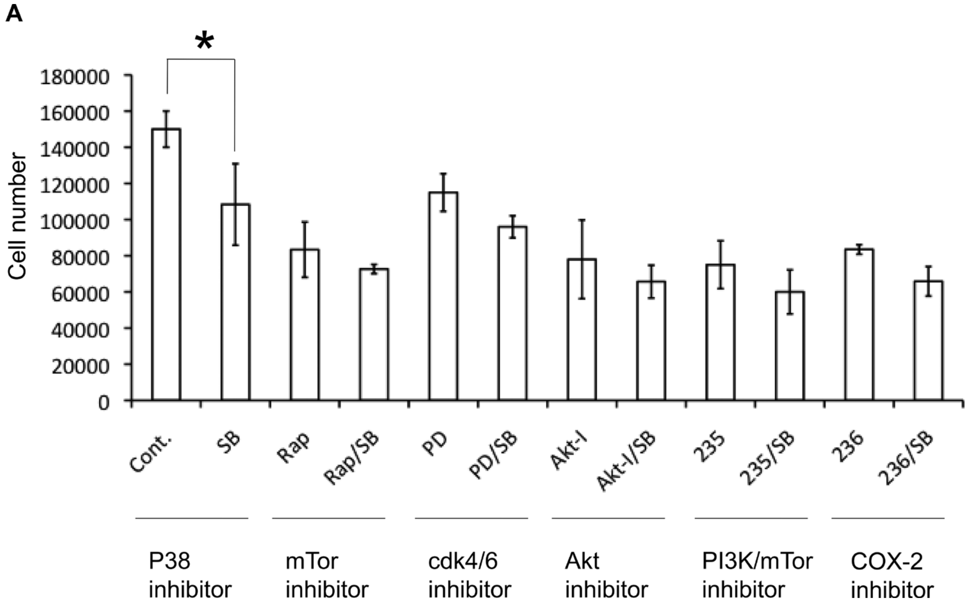

Supplementary Figure 2: Inhibition of p38 decreases cell proliferation but does not have a synergistic effect when used in combination with commercially available inhibitors of mToR, cdk5/6, Akt, dual PI3K and mToR and COX-2. Cell counting was performed by the tryphan exclusion method after incubation with inhibitors for 48 hours. The results shown in the graph are mean ± S.D. from three experiments. *$p < 0.05$. There is no difference when using p38 inhibitor in combination with these other agents.

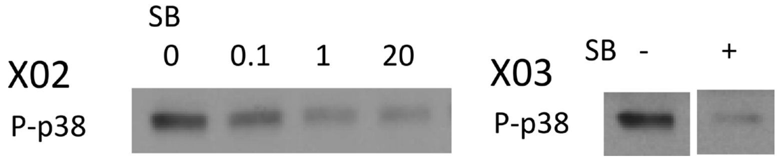

Supplementary Figure 3: Additional GSC lines (X02 and X03) were propagated with and without SB203580, an inhibitor of p38 signaling pathway. Both lines demonstrate basal activation of p38 pathway that is suppressed in a dose-dependent manner with SB203580.

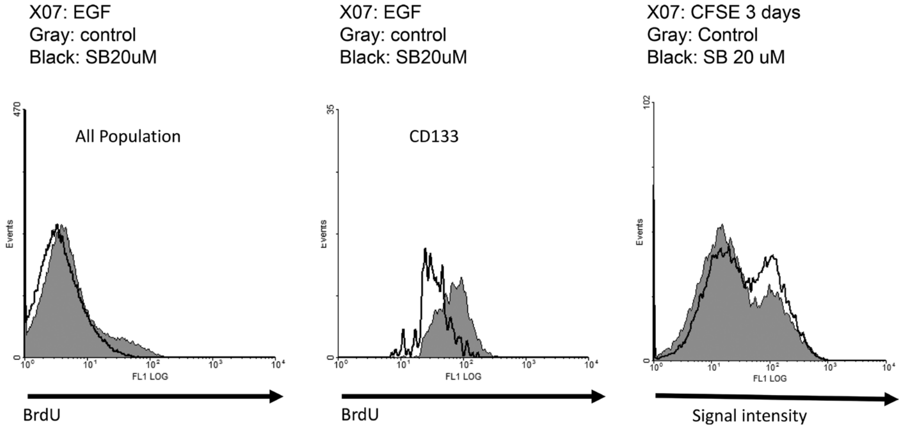

Supplementary Figure 4: Additional experiments with the X07 GSC line were performed to further replicate the results shown in Figure 3. Inhibition of the p38 pathway led to decreased BrdU incorporation in both bulk GSC population and the CD133-positive subpopulation. CFSE dye experiment (right panel) shows that p38 inhibition (solid black line) results in greater fraction of cells retaining higher signal intensity, suggesting slower cell division rate.
